# The Anti-Tumor and Immunomodulatory Effects of PLGA-Based Docetaxel Nanoparticles in Lung Cancer: The Potential Involvement of Necroptotic Cell Death through Reactive Oxygen Species and Calcium Build-Up

**DOI:** 10.3390/vaccines10111801

**Published:** 2022-10-26

**Authors:** Parul Gupta, Arpita Singh, Ajay Kumar Verma, Surya Kant, Anuj Kumar Pandey, Puneet Khare, Ved Prakash

**Affiliations:** 1 Department of Respiratory Medicine, King George’s Medical University, Lucknow 226003, Uttar Pradesh, India; 2 Department of Pharmacology, Dr. Ram Manohar Lohia, Institute of Medical Sciences, Lucknow 226010, Uttar Pradesh, India; 3 Flow Cytometry Facility, Central Instrumentation Facility, CSIR-Indian Institute of Toxicology Research, Lucknow 226001, Uttar Pradesh, India; 4 Department of Pulmonary & Critical Care Medicine, King George’s Medical University, Lucknow 226003, Uttar Pradesh, India

**Keywords:** docetaxel, plga, inflammatory response, calcium build-up

## Abstract

Taxanes, microtubule stabilizing agents, are extensively used in the treatment of non-small cell lung cancer (NSCLC). However, their clinical effectiveness remains restricted owing to significant adverse effects and drug resistance. Nanotechnology may guide chemotherapeutic drugs directly and selectively to malignant cells, improving their therapeutic efficiency. In the present study, we synthesized polylactic-co-glycolic acid (PLGA) based nanoparticles encapsulating docetaxel and evaluated their efficacy in non-small cell lung carcinoma (A549) cells and primary immune cells derived from humans. Docetaxel–PLGA nanoparticles (PLGA-Dtx) were synthesized and characterized using distinct methods. Moreover, the cytotoxicity of free docetaxel (Dtx) and Dtx-conjugated nanoparticles (PLGA-Dtx) was studied in A549 cells and peripheral blood mononuclear cells derived from humans. Furthermore, annexin V-FITC/PI staining was used to assess the mode of cell death. Additionally, human peripheral blood mononuclear cells (PBMCs) were used for assessing the associated immune response and cytokine profile following PLGA-Dtx treatment. Spherical PLGA-Dtx nanoparticles with a 150 ± 10 nm diameter and 70% encapsulation efficiency (EE) were synthesized. The MTT assay showed that the IC_50_ of PLGA-Dtx nanoparticles was significantly lower than free docetaxel in A549 cells. Cytotoxicity data also revealed the selective nature of PLGA-Dtx with no significant effects in normal human bronchial epithelial cells (BEAS-2B) and PBMCs derived from healthy donors. Interestingly, PLGA-Dtx exerted an improved effect and tempted both apoptosis and necroptosis, as evidenced by annexin V and propidium iodide–positive cells. Further, PLGA-Dtx-exposed A549 cells showed increased Cas-3, Cas-9, RIP-1, and RIP-3, indicating apoptosis and necroptosis. An increased pro-inflammatory response manifested from the enhancement of IFN-γ and TNF-α in PLGA-Dtx-exposed PBMCs, posed by the occurrence of necroptosis and the immune stimulatory effect of PLGA-Dtx. In conclusion, PLGA-Dtx has a selective anticancer potential and better immunostimulatory potential. Therefore, PLGA-Dtx may be useful for the treatment of non-small cell lung carcinoma.

## 1. Introduction

Lung cancer remains an unnerving problem in health care, as it is still the major cause of mortality worldwide. This cancer is responsible for 11.4% of newly identified cancer cases and 18% of cancer-related fatalities across all cancer types worldwide. In India, lung cancer accounts for 5.9% of all cancers and 8.1% of all fatalities, according to Global Cancer Observatory (GLOBOCAN) statistics [1]. Despite cutting-edge medical technology and advanced pathophysiological knowledge, the diagnosis and treatment of lung cancer are far from optimal. Chemotherapy is one of the most common therapeutic modalities, particularly in advanced cases. However, its application is restricted because of off-target effects, subpar pharmacokinetics, acquired drug resistance, alterations in molecular targets, activation of prosurvival cell signaling, and insufficient induction of cell death [2,3]. Therefore, development of the alternative treatment strategies which can mitigate the inefficiency of current therapies becomes indispensable. In this context, alternative delivery routes for chemotherapeutic drugs have changed the paradigm and achieved enduring responses. Different nano-based drug delivery systems have demonstrated significant benefits in the treatment of cancer, including good pharmacokinetics, specific tumor cell targeting, and reduced side effects and drug resistance [4,5]. Docetaxel (Dtx) sparked a lot of interest as a potential cancer chemotherapeutic agent, and it was approved by the FDA in 1999 for the management of nearby advanced or metastatic non-small cell lung cancer (NSCLC) [6]. Clinical studies using combinations of docetaxel and other chemotherapeutic drugs have shown improved outcomes, although the five-year survival rate for individuals with advanced lung cancer is still gloomy. Furthermore, non-selectivity and normal tissue toxicity represent an additional barrier to docetaxel’s extensive clinical use [7,8,9]. Nanotechnology has recently made impressive strides in circumventing these constraints, and scientists are actively exploring new opportunities in this area [10]. The majority of anticancer therapies have primarily focused on apoptosis, yet apoptosis resistance and immune silence have often led to treatment failure [11]. Currently, inhibitors of apoptosis protein expression are found in NSCLC [12]. Cancer cells develop apoptotic resistance by increasing or decreasing the expression of anti- or pro-apoptotic factors, respectively [13]. The mode of cell death is indispensable for ensuring an effective anticancer response to cancer cells and microorganisms that cause disease. Apoptosis is a caspase-dependent mode of cell death and has long been considered immunologically inactive while necroptosis and necrosis are assumed to elicit the immune system. In this way, inducing other types of programmed cell death, such as necroptosis, may result in greater therapeutic success and has drawn a lot of interest in the treatment of cancer. Docetaxel has been demonstrated to induce necroptotic cell death in breast cancer cells [14]. Additionally, certain nanoparticles themselves have the ability to induce or enhance programmed necrosis [11]. Even though there are still many obstacles to overcome, and practical applications are still far away, these findings have brought necroptosis into discussions as a possible method for tumor treatment.

Docetaxel has demonstrated anticancer immunostimulatory properties in addition to having direct tumoricidal effects. Preclinical and clinical studies have previously demonstrated that taxanes such as docetaxel improve antigen presentation, upregulate immune inflammation markers, and modify immune cell populations to enhance the immune response against tumors [15,16]. Docetaxel has recently been shown to decrease myeloid-derived suppressor cells (MDSCs) while simultaneously increasing their maturation into macrophages and dendritic cells, which ultimately results in increased anti-tumorigenic effects. Additionally, it has been documented that docetaxel increases the production and secretion of pro-inflammatory mediators such as IL-1, COX-2, and IL-12 [17,18]. Although the immunomodulatory effects of docetaxel have long been reported, there is less information that allows us to comprehend the influence of its nanoformulations on primary immune cells. In this context, it is important to emphasize that the efficacy against cancer may be significantly enhanced by combining anticancer cytotoxicity with immunomodulation [19,20,21]. In recent studies, researchers have also investigated the possibility that the immunomodulatory effect of nanomaterial could be used to enhance the therapeutic efficacy of encapsulated drugs [22,23,24]. Nevertheless, there are very few reports of docetaxel nanoparticles using their capacity to induce other types of cell death in addition to conventional apoptosis in lung cancer cells and their capacity to modulate the immune parameters in human primary immune cells.

In the present study, we synthesized docetaxel nanoparticles (PLGA-Dtx) and characterized them in terms of particle size, shape, and encapsulation efficiency before assessing their in vitro release patterns. Further, we examined the cytotoxicity of PLGA-Dtx in lung cancer cell lines as well as in peripheral blood mononuclear cells derived from healthy donors. Further, we investigated the mechanistic pathway that was involved in PLGA-Dtx-induced cancer cell death in addition to the immunomodulatory effect on primary immune cells. We showed that PLGA-Dtx exhibited a more potent anticancer effect in A549 cells compared with the effect of the free drug docetaxel. Further, selective uptake of PLGA-Dtx nanoparticles was demonstrated in A549 cells, whereas normal human bronchial epithelial cells (BEAS-2B) showed significantly reduced uptake. PLGA-Dtx exhibits a potent inducing effect on both apoptotic and necroptotic signaling, with the former occurring through a caspase-dependent mechanism and the latter occurring via a RIP-1/RIP3/MLKL pathway. Additionally, PLGA-Dtx induced a vigorous pro-inflammatory response with significant increases in IFN-γ and TNF-α cytokine concentrations. We also examined the role of reactive oxygen species and intracellular calcium levels in PLGA-Dtx-induced cell death.

## 2. Materials and Methods

### 2.1. Materials

Docetaxel, poly (lactic-co glycolic acid), polyvinyl alcohol, triethylamine, HPLC grade water, DSPE-PEG, DMEM, DMEM/F-12, Roswell Park Memorial Institute medium (RPMI), 3-[4,5-dimethylthiazol-2-yl]-2,5 diphenyl tetrazolium bromide (MTT), dimethyl sulphoxide (DMSO), Histopaque for PBMCs isolation, DCFDA, JC-1, and fura-2 AM dye were purchased from Sigma Chemical Co., Ltd. (Burlington, MA, USA). Phosphate buffered saline (PBS), antibiotic–antimycotic solution, and fetal bovine serum (FBS) were procured from Gibco, Invitrogen Cor. (Waltham, MA, USA). American Type Culture Collection (ATCC) supplied the A549 (human lung adenocarcinoma cell line) and BEAS-2B (normal human bronchial epithelial cells) for this study (Manassas, VA, USA). Necroptosis inhibitor necrostatin-1 (Nec-1), apoptosis inhibitor Z-VAD-fmk, primary antibodies, such as caspase-3, caspase-8, caspase-9, Bcl-2, Bax, β-actin, RIPK1, RIPK3, and MLKL were procured from Santa Cruz (Santa Cruz, CA, USA). Cleaved caspase-3, cleaved caspase-9, and cleaved caspase-8 were procured from Cell Signaling Technology (Danvers, MA, USA). Cell-culture-grade plasticware was purchased from Genetix Biotech Asia Pvt. Ltd. The FITC Annexin V Apoptosis Detection Kit, Human Th1/Th2/Th17 CBA kit for cytokine analysis, antibodies allophycocyanin (APC)-Cy7 conjugated anti-CD4, FITC-conjugated anti-CD-8, and Alexa flour 700-conjugated anti-CD19 for immunophenotyping were purchased from BD Biosciences (Franklin Lakes, NJ, USA). 

### 2.2. Preparation of PEGylated PLGA-Dtx Nanoparticles

PEGylated PLGA-Dtx nanoparticles were prepared in the same way that our group reported in a previous study [25]. The simple nanoprecipitation method was used to make the PEGylated PLGA nanoparticles encapsulating docetaxel. Briefly, PLGA and DSPE-PEG were combined in chloroform at a weight ratio of 1:1. The solvent was removed using a moderate air stream, and the resulting film was then redissolved in 250 µL of dichloromethane. A known amount of docetaxel (75 µL from docetaxel stock of 10 mg/mL in DMSO) was then added to the above-mentioned mixture and sonicated for 15 s. Then, 0.4 mL of an aqueous polyvinyl alcohol (PVA) solution with 2.5% PVA was added while the mixture was being stirred. The resultant thick emulsion was further sonicated for 30 s before being added to 10 mL of 0.3% aqueous PVA solution while vigorously swirling. PEGylated PLGA-Dtx NPs were created as a consequence of the reaction mixture having an optically transparent appearance. The leftover surfactant and other unreacted compounds were removed by dialysis against deionized water, using cellulose membranes of 12–14 kDa. The nanoparticles were then sterilized filtered after dialysis and kept at 4 °C. Following the sterile filter, the resultant NPs were lyophilized and stored in powder form.

### 2.3. Characterization of PLGA-Dtx Nanoparticles

#### 2.3.1. Dynamic Light Scattering (DLS)

The DLS was used to determine the zeta potential (ZP), polydispersion index (PDI), and particle size of the PLGA-Dtx NPs. DLS analysis was carried out with a Zetasizer Nano-ZS that was outfitted with a 4.0 mW, 633 nm laser (Model 168 ZEN3600, Malvern Instruments Ltd., Malvern, UK).

#### 2.3.2. Atomic Force Microscopy (AFM)

AFM was used to investigate the PLGA-Dtx NPs in terms of their size, shape, and surface morphology. For AFM analysis, PLGA-Dtx NPs were diluted in deionized water to the desired concentration and then sonicated for 30 s. A total of 50 µL of the sonicated sample was placed on the substrate (mica) and analyzed using a WITec Atomic Force Microscope alpha300 A (WITec GmbH, Ulm, Germany).

#### 2.3.3. Transmission Electron Microscopy (TEM)

PLGA-Dtx nanoparticles were characterized for their shape and size using TecnaiTM G2 Sprit (FEI, The Netherlands) transmission electron microscope (TEM). The suspension of PLGA-Dtx NPs was added to a carbon-coated copper grid for TEM analysis.

### 2.4. Encapsulation Efficiency (%EE) of PLGA-Dtx Nanoparticles

Reverse-phase HPLC method was used to quantify the amount of docetaxel in the PLGA nanoparticles according to the method reported by Koopaei et al. [26]. To summarize, lyophilized PLGA-Dtx nanoparticles weighing 2.5 mg were first dissolved in 1 mL of acetonitrile, then the mixture was softly agitated before being subjected to sonication for 6 min. The polymer was then precipitated by adding 2 mL of methanol to the mixture. After that, the sample was filtered, and HPLC analysis was used to quantify the amount of docetaxel that was present in the filtrate. HPLC analysis was carried out with a Waters LC module (Waters Associates, Vienna, Austria) equipped with a reversed-phase C18 column. The UV detector was calibrated to 230 nm, and an isocratic mobile phase consisting of acetonitrile and water at a volumetric ratio of 65:35 was utilized at a flow rate of 1 mL per minute. The encapsulation efficiency was measured by comparing the amount of Dtx that was trapped in nanoparticles to the amount of Dtx that was used initially to make nanoparticles (75 µL from docetaxel stock of 10 mg/mL in DMSO). Encapsulation efficiency is expressed as a percentage and was calculated by:

Encapsulation efficiency (EE%) = (Wt/Wi) × 100%

where Wt is the total amount of drug in the nanoformulation and Wi is the total quantity of drug added initially during preparation.

### 2.5. Release of Dtx from PLGA-Dtx Nanoparticles

The release of Dtx from PLGA-Dtx was assessed in vitro according to the method reported by Koopaei et al. [26]. The in vitro release study of docetaxel from PLGA nanoparticles was performed by the dialysis bag method at pH 7.4 and 37 °C temperature conditions. Briefly, PLGA-Dtx nanoparticles (2.5 mg) suspended in isotonic PBS solution (pH 7.4) were placed in a dialysis bag. After that, the dialysis bag was submerged in 50 mL of PBS solution, and the whole device, which had been wrapped and sealed with parafilm, was put on a rotating shaker and kept at 37 °C for three days. At each specific time point, 2 mL of solution was removed and recouped with an equal amount of fresh buffer to maintain the perfect sink condition. To determine the Dtx concentration, collected solution was quantified using HPLC. Drug release was determined using the following equation:

Amount of drug released mg/mL = concentration × dissolution bath volume × dilution factor/1000.

Drug release (%) = volume of sample withdrawn (mL)/bath volume (v) × P (t − 1) + Pt

where Pt = percentage release at time t. Where P (t − 1) = percentage release previous to ‘t’.

### 2.6. Cell Line and Culture Conditions

Human lung alveolar adenocarcinoma (A549) cells and normal human lung bronchial epithelial (BEAS-2B) cells were used for the experiments. The A549 cell line is a widely used human lung adenocarcinoma cell line that was derived from a primary lung tumor, whereas the BEAS-2B cell line was originally established as a human non-tumorigenic lung epithelial cell line derived from human lung tissue. A549 cells were cultured in T75 flasks in DMEM F-12 culture media that was supplemented with 10% fetal bovine serum, 0.2% sodium bicarbonate, and antibiotic–antimycotic solution (10 mL/L). The flasks were kept at 37 °C in an environment containing 5% carbon dioxide and a humidified atmosphere. BEAS-2B cells were cultured in T75 flasks in RPMI culture media as mentioned above. After reaching the confluency of 80 to 90%, cells were trypsinized using 0.25% trypsin-EDTA solution and then cultured in 96 well culture plates. Experiments were performed in triplicate and repeated three times.

Human PBMCs were obtained from heparinized peripheral blood through density gradient centrifugation using Ficoll (Histopaque). Cells were cultured in RPMI 1640 medium supplemented with 10% heat-inactivated FBS, HEPES buffer, and 1% antimycotic solution in a humidified chamber. Blood samples were collected from five healthy donors and five newly diagnoses lung cancer patients before the start of any treatment during standard diagnostic evaluations from the Department of Respiratory Medicine, King George’s Medical University, Lucknow (2018–2019). The study was approved by the ethical advisory board and the ethical approval number was 86th ECM II BThesis/P22. All subjects gave written informed consent as per the Declaration of Helsinki.

### 2.7. Cell Viability/Death Assay

The MTT [3-(4,5-dimethylthiazol-2-yl)-2,5-diphenyltetrazolium bromide] assay was performed to assess the viability of A549 cells, BEAS-2B cells, and PBMCs, when cultured alone or in treatment with Dtx/PLGA-Dtx as reported earlier [25]. Briefly, 5000 viable cells were plated in 96 well cell culture plates in 200 µL of medium and incubated with varying concentrations (0.025 µg/mL–1 µg/mL) of the drug docetaxel alone and with PLGA-Dtx nanoconjugate containing the same amount of drug as equivalent to the free drug for 24 h. After incubation for the desired time, 10 µL of MTT solution was added to each well and waited for the next 3 h. The cell viability was assessed at 550 and 660 nm utilizing a microplate reader.

### 2.8. Cellular Internalization Assessment

Flow cytometry-based evaluation of PLGA-Dtx internalization in cultured cells was performed in accordance with the established technique. Cells were plated in a 12-well culture plate at a concentration of 1 × 105 cells/mL/well. Cultured cells were treated with IC50 value of PLGA-Dtx for different time points up to 6 h. After completion of the desired exposure time, cells were trypsinized using 0.25% trypsin and centrifuged at 1200 rpm for 10 min. Supernatant was removed and the pellet was resuspended in 1X PBS (1 mL). The uptake of particles was determined by flow cytometer (FACS INFLUX). For uptake inhibitor, study cells were pretreated with 10 µg/mL of amiloride, 10 µg/mL of chlorpromazine, 5 µg/mL of genistein, and 5 µg/mL of filipin prior to PLGA-Dtx treatment for 24 h. The uptake of particles was determined by flow cytometer (FACS Canto) as mentioned above.

### 2.9. Annexin V/PI Assay

Apoptosis in A549 cells was analyzed using the Annexin V-FITC Apoptosis Staining/Detection Kit. Cells were treated with Dtx and PLGA-Dtx nanoconjugate for 24 hr. After completion of exposure time, analysis of apoptosis was performed according to the manufacturer’s protocol.

### 2.10. Western Blot Analyses

Western blot analysis was performed according to previously published study by Roy et al. [27].

### 2.11. Measurement of ROS Generation and Oxidative Stress Markers

A549 cells (1 × 10^5^ cells/well) were cultured in a 12-well black flat-bottom cell culture plate. Cells were treated with Dtx/PLGA-Dtx nanoconjugate time-dependently up to 24 h. After desired incubation, cells were washed two times by suspending in PBS. Afterward, 20 µM of 2,7′-dichlorodihydrofluorescein diacetate (H2DCFDA) dye was added to each well. The plate was further incubated for 30 min and after completion of the incubation period, medium was discarded. A total of 200 µL PBS was added and fluorescence was analyzed by a flow cytometer. To study the effect of PLGA-Dtx nanoparticles on various oxidative stress markers, A549 cells were treated with PLGA-Dtx for 24 h. Reduced glutathione (GSH) content was assayed in the cells’ homogenate according to the method reported by Ellman [28]. Lipid peroxidation (LPO) in the cell homogenate was determined by estimating the formation of malondialdehyde (MDA) [29]. Protein carbonyl content was measured in the homogenate according to the method reported by Levine et al. [30]. Catalase activity was determined by the method reported by Sinha [31]. Glutathione S transferase (GST) activities were assayed according to the method reported by Moron [32].

### 2.12. Determination of Mitochondrial Membrane Potential (MMP) and Cytosolic-Free Calcium

Mitochondrial membrane potential was examined using the mitochondrial staining dye JC-1. A549 Cells were treated with PLGA-Dtx nanoconjugate and inhibitors for 12 h in a 12-well cell culture plate. Cells were washed with PBS two times and further incubated with 0.3 μM JC-1 dye for 30 min. After completion of the incubation period, cells were washed two times with PBS and again suspended in 500 μL of PBS. Fluorescence intensity was measured using a flow cytometer. Intracellular Ca^2+^ level was assessed by the fluorescence intensity ratio of the calcium probe fura-2 AM. Briefly, the cells were treated with PLGA-Dtx nanoconjugate and inhibitors for 24 h in a 12-well cell culture plate. Cells were washed with PBS two times and further incubated with 0.5 μM fura-2 AM dye for 30 min. After completion of the incubation period, cells were washed two times with PBS and again suspended in 500 μL of PBS. Fluorescence intensity was measured using a flow cytometer.

### 2.13. Immunophenotyping of PBMCs and Cytokine Analysis

PBMCs (2 × 10^6^ cells/mL) from healthy donors were harvested in 24 well culture plates. Cells were treated with a non-cytotoxic concentration of PLGA-Dtx as analyzed by MTT assay for 72 h for identification of B cell and T cell populations as described by Yadav et al. [33]. The cytokine measurement was performed on the supernatant of the cultured PBMCs by using a TH1/TH2/TH17 cytometric bead array (CBA) kit. Samples were prepared for cytokine analysis as per the manufacturer’s instructions and analyzed on the same day by a flow cytometer (BD INFLUX).

### 2.14. Statistical Analysis

Data were expressed as means ± SEM. Statistical analysis was performed using one-way or two-way ANOVA for comparison between groups. *p* < 0.05 was set as statistical significance. GraphPad Prism 6 (GraphPad Software Inc., San Diego, CA, USA) was used throughout the study for statistical analysis.

## 3. Results

### 3.1. Preparation and Characterizations of PLGA-Dtx

**Figure 1:**
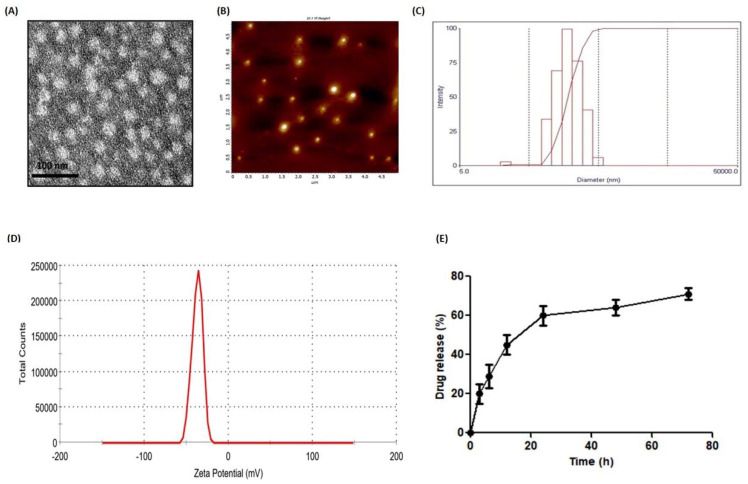
Characterization and drug release profile of PLGA-Dtx nanoparticles. (**A**) TEM image; (**B**) AFM image; (**C**) mean particle size distribution; (**D**) zeta potential; (**E**) in vitro release profile of PLGA-Dtx NPs.

PLGA nanoparticles encapsulating docetaxel (PLGA-Dtx) were prepared through a simple nanoprecipitation method with some modest changes from the previously reported method. Docetaxel and PLGA were stabilized and solubilized using polyvinyl alcohol (PVA) and polyethylene glycol (PEG), which led to the formation of spherical nanoparticles within a stable emulsion. The TEM and AFM images of PLGA-Dtx, as shown in Figure 1A,B, show a nearly spherical structure. The mean diameter of nanoparticles and zeta potential were measured by DLS and found to be 150 ± 10.2 nm and −37 ± 0.7 mV, respectively (Figure 1C,D). The polydispersity index (PDI) of PLGA-Dtx nanoparticles was 0.157 and they had a narrow size distribution. 

The drug encapsulation efficiency was determined as 60–70% using high-performance liquid chromatography (HPLC). The in vitro release of docetaxel from PLGA nanoparticles displayed a typical burst release, followed by a slow and sustained release for three days. As shown in Figure 1E, around 30 to 40% of the total encapsulated docetaxel was released within 24 h. A sustained release of docetaxel was observed from the PLGA nanoparticles and around 80% was released in three days. 

### 3.2. In Vitro Cytotoxicity Evaluation of PLGA-Dtx Nanoparticles

**Figure 2:**
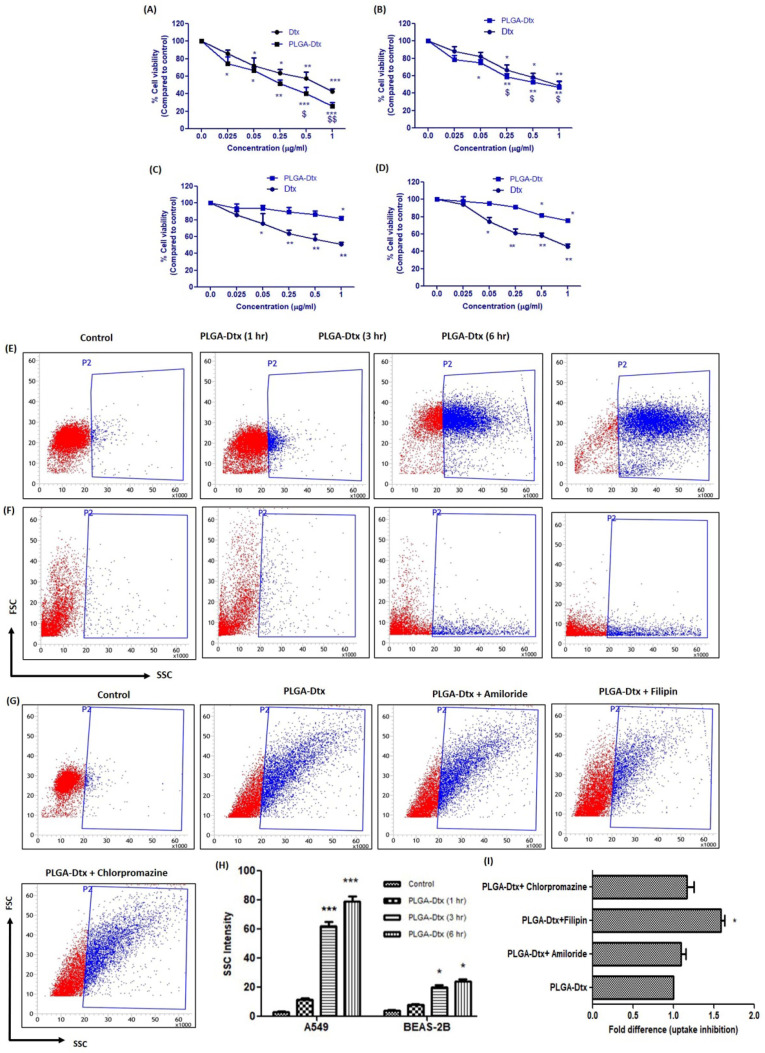
In vitro cellular cytotoxicity and uptake assay. (**A**) In vitro cytotoxicity of free Dtx and PLGA-Dtx NPs against A549 cancer cells; (**B**) PBMCs derived from lung cancer patients; (**C**) BEAS-2B cells; (**D**) PBMCs derived from healthy donors; Flow cytometer analysis of in vitro cellular uptake of PLGA-Dtx nanoparticles for 3 and 6 h in (**E**) A549 cancer cells; (**F**) BEAS-2B cells; (**G**) effect of pharmacological inhibitors on uptake of PLGA-Dtx in A549 cancer cells; (**H**) graphical representation of in vitro cellular uptake (**I**) graphical representation of effect of pharmacological inhibitors. Data were the mean ± SEM from at least three independent experiments. (* *p* < 0.05, ** *p* < 0.01, and *** *p* < 0.001 compared with the control group), ($ *p* < 0.05 and $$ *p* < 0.01 compared with the free Dtx).

As shown in Figure 2A,B, PLGA-Dtx induced dose-dependent cytotoxicity at 24 h with a compelling effect on A549 and PBMCs derived from lung cancer patients, with IC_50_ values of 0.23 ± 0.04 and 0.49 ± 0.08 µg/mL, respectively, as compared with free docetaxel (Dtx), with higher IC_50_ values of 0.54 ± 0.02 and 0.68 ± 0.04 µg/mL, respectively. We also examined the effects of Dtx and PLGA-Dtx on A549 cells at 48 h (Figure S1) and found dose dependent toxicity. Further experiments using non-carcinoma cell lines BEAS-2B and healthy donors’ PBMCs at 24 h, as shown in Figure 2C,D, showed that PLGA-Dtx induced cytotoxicity at a much higher concentration as compared with Dtx, with higher IC_50_ values of 3.65 ± 0.24 and 2.75 ± 0.14 µg/mL, respectively, as compared with Dtx with lower IC_50_ values of 0.79 ± 0.02 and 0.58 ± 0.02 µg/mL, respectively. These results show that PLGA-Dtx was more selective, being more toxic towards cancer cells with relatively less toxicity in non-carcinoma cells.

### 3.3. Cellular Uptake of PLGA-Dtx NPs

To understand the discerning effects of PLGA-Dtx on cancer cells, the uptake of NPs by cells must be better characterized. For this, we exposed A549 and BEAS-2B cells to PLGA-Dtx at different time points. As indicated by Figure 2E,F, side scatter intensity increased time-dependently in both cells, whereas the intensity in A549 cells was much higher than in BEAS-2B cells. The selective cytotoxicity effect may be partially explained by these results demonstrating the uptake behavior in both carcinoma and non-carcinoma cells. Moreover, to understand the mechanism of uptake in cancer cells, we treated A549 cells with PLGA-Dtx in the presence of different pharmacological inhibitors of endocytosis. We did not include the phagocytosis inhibitor as this process is particular to certain immune cells. Results shown in Figure 2G indicate that filipin, an inhibitor of caveolae-mediated processes, markedly inhibited the uptake of PLGA-Dtx, whereas the uptake was unaffected by treatment with amiloride, a micropinocytosis inhibitor, and chlorpromazine, a clathrin-mediated endocytosis inhibitor. These findings suggest that the uptake of PLGA-Dtx may involve a caveolae-mediated uptake pathway.

### 3.4. Assessment of Cell Death Mechanism of PLGA-Dtx

**Figure 3:**
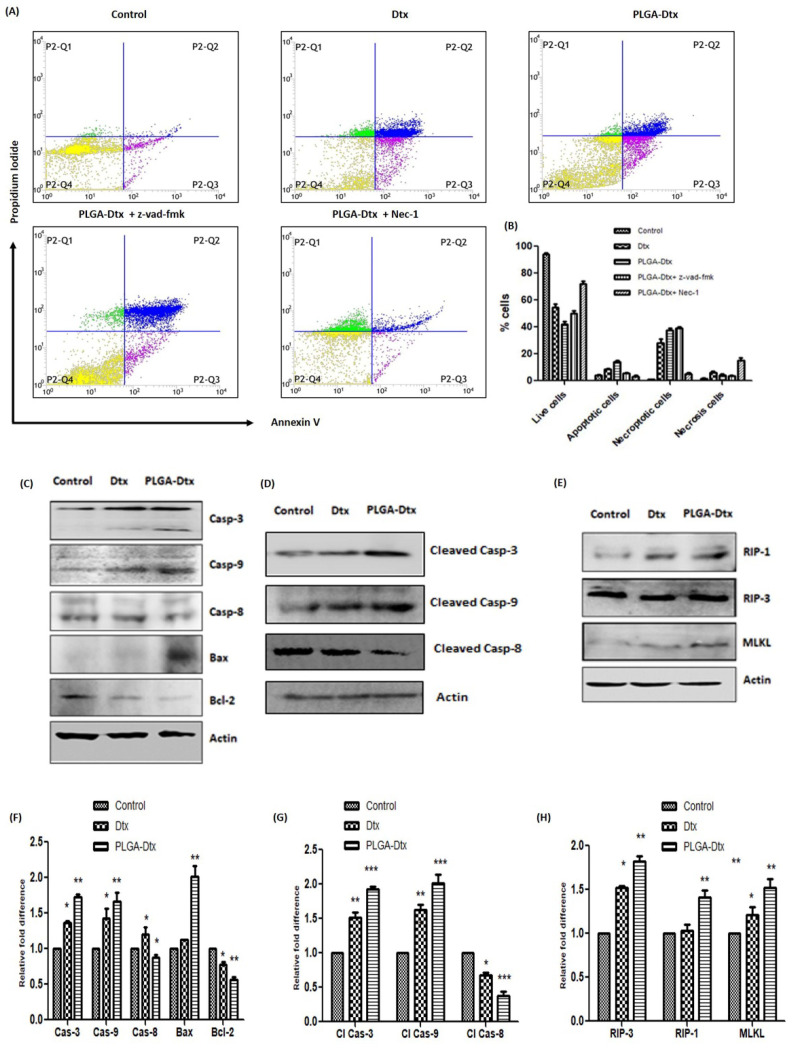
Flow cytometry analysis of lung cancer cell death mode caused by Dtx/PLGA-Dtx. (**A**) Influences of Z-VAD-fmk and Nec-1 on apoptotic and necroptotic cells, respectively. A549 cells were pretreated with 20 μM Z-VAD-fmk or 50 μM Nec-1 for 30 min and subsequently treated with IC_50_ value of PLGA-Dtx and relative dose of Dtx for 24 h; (**B**) representative bar diagram to show the percentage of cell death including live cells, apoptotic cells, late apoptosis/necroptosis, and necrosis; influences of Dtx and PLGA-Dtx on apoptotic and necroptotic signaling proteins. A549 cells were treated with IC_50_ value of PLGA-Dtx and relative dose of Dtx for 24 h and cell lysates were analyzed by western blot with indicated antibodies. (**C**) apoptotic proteins; (**D**) cleaved caspases; (**E**) necroptotic proteins; (**F**–**H**) representative bar diagram showing fold difference in apoptotic and necroptotic protein levels. Data were the mean ± SEM from at least three independent experiments. (* *p* < 0.05, ** *p* < 0.01, and *** *p* < 0.001 compared with the control group.).

Apoptosis has long been implicated in chemotherapeutic drug-induced cell death. We used annexin V/propidium iodide dual staining to assess apoptosis/necrosis in A549 cells. Apoptosis starts with the loss of the plasma membrane with exposure to the outer part of the plasma membrane. When phosphatidylserine is exposed to the outer leaf of the plasma membrane, the annexin V dye attaches to the cells and so can be used to identify apoptosis. The percentage of apoptotic and late apoptosis/necroptotic cells was significantly increased following the exposure of PLGA-Dtx for 24 h, as depicted from the histograms in Figure 3A. PLGA-Dtx significantly enhanced early apoptosis up to 15 ± 2.02% compared with the control (4.4 ± 1.1%). 

Consistently, PLGA-Dtx upregulated late apoptosis/necroptosis (36 ± 3.4%) as compared with the control (1.2 ± 0.04%). However, as suggested by the cell toxicity data, the effect of Dtx was less pronounced in A549 cells. The apoptotic and necroptotic cell percentages in Dtx-treated cells were 8.1 ± 1.4 and 15± 2.7%, respectively. Further, we assessed the implication of apoptosis and necroptosis regulation by PLGA-Dtx in lung cancer cells. To prevent the induction of apoptosis by PLGA-Dtx, we treated A549 cells in the presence of Z-VAD-fmk, which blocks apoptosis by caspase inhibition. To our surprise, inhibition of apoptosis switched the cells to necroptosis as the percentage of necroptotic cells was increased (42 ± 2.4%). As expected, apoptosis inhibition by Z-VAD-fmk resulted in a decreased percentage of apoptotic cells. In addition, necroptosis inhibitor Nec-1 partially abrogated both necroptotic and apoptotic cells. These findings propose the involvement of both apoptosis and necroptosis in PLGA-Dtx-induced cell death. To confirm our findings from the annexin V/PI assay, we analyzed the proteins involved in both apoptosis and necroptosis processes by western blotting. As shown in Figure 3C, PLGA-Dtx significantly increased the level of caspase-3 and caspase-9, whereas the level of caspase-8 was decreased. In addition, PLGA-Dtx also upregulated the proapoptotic protein Bax and downregulated the antiapoptotic protein Bcl-2. For further validation of involvement of apoptosis, we also analyzed the levels of cleaved caspases. As shown in Figure 3D, similarly to pro-caspase levels, cleaved caspase-3 and cleaved caspase-9 were upregulated contrarily to cleaved caspase-8, which was downregulated in PLGA-Dtx-treated cells. Concurrently, as shown in Figure 3E, PLGA-Dtx also enhanced the levels of necroptotic proteins RIP-1, RIP-3, and MLKL. Taken together, our results demonstrate the involvement of both cell death pathways.

### 3.5. Involvement of ROS and Calcium Accumulation in PLGA-Dtx-Induced Cell Death

**Figure 4:**
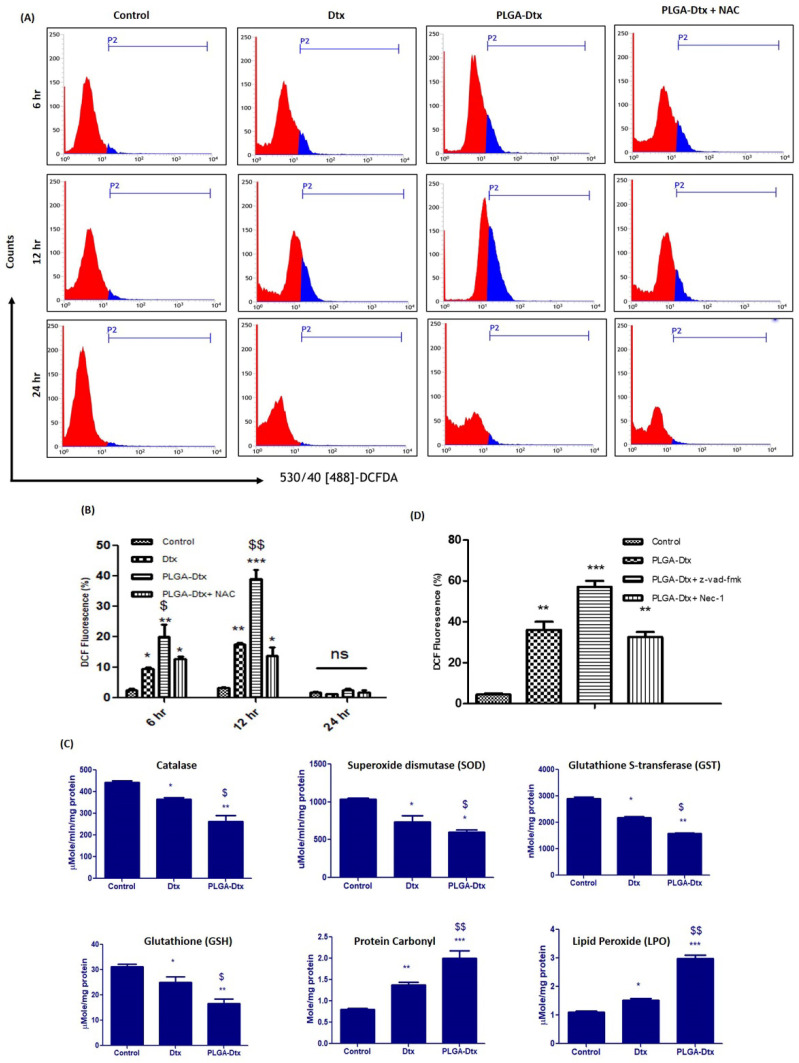
ROS production resulting from exposure to PLGA-Dtx in A549 cells. (**A**) Cells were pretreated with antioxidant NAC for 30 min and subsequently treated with IC50 value of PLGA-Dtx for different periods (6, 12, and 24 h), and then intracellular ROS was measured by DCFH2-DA fluorescence; (**B**) representative bar diagram to show the change in DCF fluorescence; (**C**) effect of Dtx and PLGA-Dtx exposure on antioxidant enzymes and oxidative stress markers in A549 cells; (**D**) representative bar diagram for showing the effect of Z-VAD-fmk and Nec-1 on ROS production. Data were the mean ± SEM from at least three independent experiments. (* *p* < 0.05, ** *p* < 0.01, and *** *p* < 0.001 compared with the control group.), ($ *p* < 0.05 and $$ *p* < 0.01 compared with the free Dtx).

We next investigated the potential role of reactive oxygen species (ROS) in the regulation of apoptosis and necroptosis induced by PLGA-Dtx. We used cell-permeable 2’,7’-dichlorodihydrofluorescein diacetate (H2DCFDA) dye for the detection of reactive oxygen species. The non-fluorescent H2DCFDA was changed into the fluorescent 2’,7’-dichlorofluorescein via oxidation and intracellular esterases cleaving the acetate groups. Flow cytometer analysis of ROS depicted in Figure 4A showed that PLGA-Dtx induced markedly enhanced ROS production compared with control as stipulated by a right shift of the DCFDA peak. 

NAC is a widely used antioxidant and pretreatment with NAC abrogated the induced ROS. Intracellular antioxidant enzyme systems tightly regulate ROS production, and the amount of these antioxidant enzymes is either raised or lowered to shield cells from harmful ROS. We, therefore, assessed the level of expression of necessary antioxidant enzymes in the cells following exposure to the PLGA-Dtx treatment.

While protein carbonyl and lipid peroxidation were found to be elevated following the treatment with PLGA-Dtx, the expression of catalase, superoxide dismutase-1 (SOD-1), GST, and GSH was suppressed (Figure 4C). RIPK1 has been acknowledged to contribute in the production of ROS and also play crucial roles in the regulation of cell survival and death. Therefore, we assessed the role of necroptosis and apoptosis in ROS generation from PLGA-Dtx-treated cells. To analyze the roles of necroptosis and necroptosis in PLGA-Dtx-induced ROS production, A549 cells were treated in presence of Z-VAD-fmk and Nec-1. As evident from Figure 4D, Nec-1 reduced PLGA-Dtx-induced ROS generation, whereas Z-VAD-fmk treatment increased it. The results suggest that necroptosis was important for regulating ROS homeostasis. 

**Figure 5:**
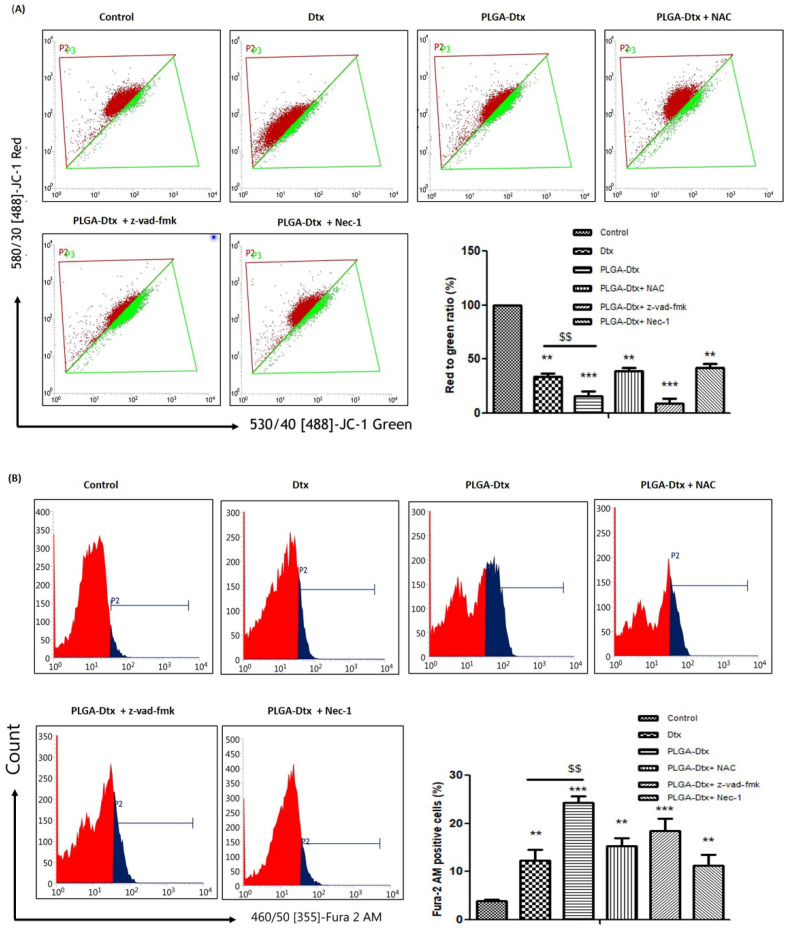
PLGA-Dtx-induced accumulation of reactive oxygen species is accompanied by mitochondrial depolarization and calcium build-up. (**A**) A549 cell lines were pretreated with the free radical scavenger NAC or Z-VAD-fmk or Nec-1 and subsequently treated with IC_50_ value of PLGA-Dtx and relative dose of Dtx for 12 h and then stained with JC-1 to analyze loss of ΔΨm. In control cells, JC-1 dye accumulates in mitochondria and forms red fluorescent J-aggregates, reflecting high mitochondrial membrane potential, whereas green cytosolic fluorescence indicates low mitochondrial membrane potential; (**B**) A549 cell lines were pretreated with the free radical scavenger NAC or Z-VAD-fmk or Nec-1 and subsequently treated with IC_50_ value of PLGA-Dtx and relative dose of Dtx for 12 h and then stained with fura-2 AM dye to analyze the intracellular calcium level; increased intensity of fura-2 AM fluorescence showing the intensity of intracellular calcium levels. Data were the mean ± SEM from at least three independent experiments. (** *p* < 0.01, and *** *p* < 0.001 compared with the control group), ($$ *p* < 0.01 compared with the free Dtx).

Because high ROS generation is connected to mitochondrial malfunction, we investigated whether mitochondria membrane potential played a role in PLGA-Dtx-induced A549 cell death. Dissipated mitochondria potential (ΔΨm) has been used as a marker in cell death and ROS overproduction. We used JC-1 dye for analyzing the dissipation in mitochondria potential. JC-1 dye is a lipophilic, cationic dye that enters normal cells’ mitochondria and forms reversible J-aggregates. However, as membrane potential drops in response to cell damage, JC-1 monomers are produced resulting in green fluorescence. Our findings from Figure 5A show that treatment with PLGA-Dtx led to depolarization of the mitochondrial membrane as green fluorescence was increased in PLGA-Dtx-treated cells compared with the control. This suggests that JC-1 monomer was abundant, which is suggestive of MMP being depleted. In addition, the membrane potential was recovered by Nec-1 therapy but not by Z-VAD-fmk treatment. In addition, we demonstrated the role of intracellular calcium build-up in PLGA-Dtx-induced cell death. To assess the potential role of calcium in the induction of PLGA-Dtx-mediated necroptosis, fura-2 AM staining was used. Fura-2 AM is a fluorescent dye that binds to intracellular calcium. According to the results shown in the figure, PLGA-Dtx exposure significantly increased intracellular Ca^2+^ levels in A549 cells (Figure 5B). We thus came to the conclusion that ROS overproduction, mitochondrial malfunction, and calcium accumulation are all intimately associated with PLGA-Dtx-induced cell death.

### 3.6. Immunomodulatory Effects of PLGA-Dtx on Peripheral Blood Mononuclear Cells

**Figure 6:**
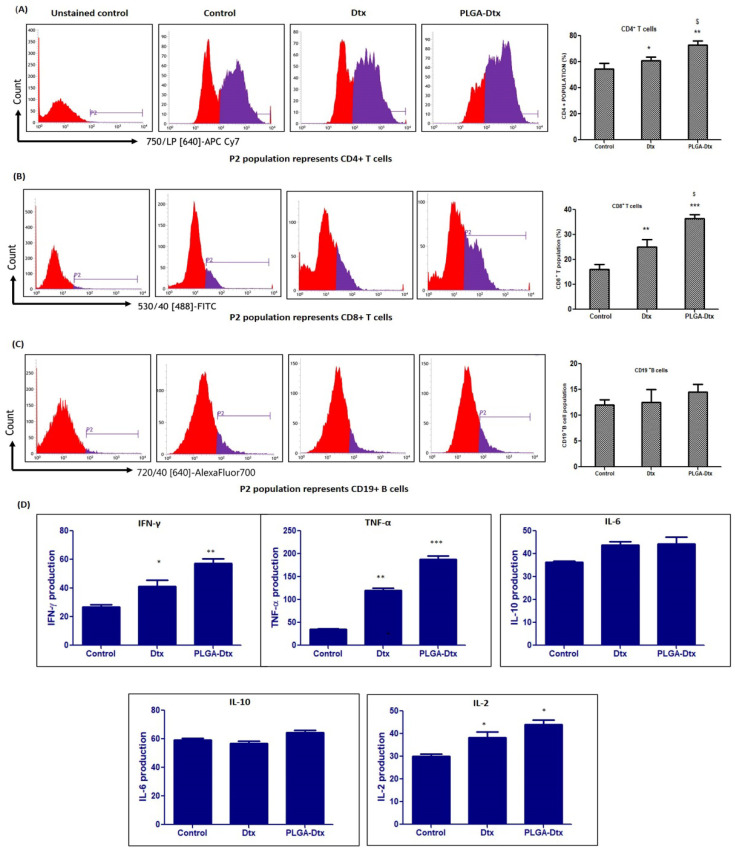
Flow cytometric analysis of effect of Dtx and PLGA-Dtx on the relative distribution of T lymphocyte (CD4+/CD8+) and B lymphocyte (CD19+) populations in Con A (T cell activation) and LPS (B cells activation) stimulated peripheral blood mononuclear cells. PBMCs were treated with non-cytotoxic concentration of PLGA-Dtx derived from MTT assay for 72 h. (**A**) Histogram showing the CD4+ T lymphocytes cells; (**B**) histogram showing the CD8+ T lymphocytes cells; (**C**) histogram showing the CD19+ B lymphocytes cells; (**D**) effect of Dtx and PLGA-Dtx in supernatant of PBMCs treated with Dtx and PLGA-Dtx for 24 h. Data were the mean ± SEM from at least three independent experiments. (* *p* < 0.05, ** *p* < 0.01, and *** *p* < 0.001 compared with the control group), ($ *p* < 0.05 compared with the free Dtx).

We pursued the role of necroptosis in the anti-tumor response after establishing the role of necroptosis in cell death induced by PLGA-Dtx, as necroptosis can elicit sturdy adaptive immune responses that may defend against tumor progression. In order to assess functional immune responses, a less cytotoxic dose of PLGA-Dtx was selected based on the cytotoxicity data from the MTT assay. Immunophenotyping studies (CD3e+, CD4+ cells, CD8+ T, and CD19 B cells) were conducted on ex vivo PBMCs from healthy donors to examine the impact of PLGA-Dtx on particular lymphocyte populations. In comparison to the control group, PLGA-Dtx treatment resulted in significantly higher relative levels of CD3e+ T cells. The CD3e+ population was further gated and examined for CD4+ and CD8+ T cells. PBMCs treated with PLGA-Dtx displayed a notably increased proportion of CD8+ T and CD4+ T cells (Figure 6A,B). Further analysis revealed that PLGA-Dtx had no substantial effect on CD19+ B cells (Figure 6C). Next, we assessed the various levels of cytokines secreted by PBMCs in order to clarify the immunomodulatory effects. As suggested by Figure 6D, the IFN-γ and TNF-α cytokines were more highly expressed in the PLGA-Dtx-treated cells when compared with the control group. In contrast, PLGA-Dtx had no impact on the expression of the IL-10 and IL-6 genes when compared with the control group. The level of IL-2 was slightly increased in PLGA-Dtx-treated cells when compared with the control group.

## 4. Discussion

Chemotherapeutic drugs have substantial limitations, such as limited water solubility and systemic toxicity, despite their potency against a number of tumor forms [34]. To address these challenges and more effectively deploy anticancer drugs in cancer therapy, researchers have concentrated their efforts on the creation of nanoformulations for anticancer drug delivery systems. Taxanes have earned a reputation in the last decade as prospective cancer chemotherapeutic agents. These compounds were first identified as mitotic inhibitors, and their anti-neoplastic properties were attributed to their capacity to prevent cellular division by stabilizing microtubules. Different nanoformulations of docetaxel have been produced in order to increase its solubility, bioavailability, and pharmacokinetic properties [10]. Docetaxel nanoparticles have primarily been studied for their ability to lower “off-targeted” toxicity, although their effects on various modes of cell death and their immunomodulatory potential have yet to be fully explored. In the present study, we first synthesized and characterized the PLGA nanoparticles encapsulating the drug docetaxel. In addition, we demonstrated promising selective anticancer effects of these docetaxel nanoparticles (PLGA-Dtx) against A549 cells and PBMCs. The findings of this study concluded that, when compared with free docetaxel, PLGA-Dtx can both trigger the process of selective cell death and have potent immunomodulatory effects. Recent studies have proved that docetaxel acts as an immune stimulator. Moreover, we revealed that PLGA-Dtx induced cell death in cancer cells through pleiotropic mechanisms. 

The nanoprecipitation process that was used in the synthesis of nanoparticles results in PLGA nanoparticles with PEG-decorated surfaces. The use of DSPE-PEG (phospholipid) in our preparation of nanoparticle synthesis provides more stability in the aqueous environment. In previous studies, the lipid–PLGA combination was used to fabricate composite PLGA nanoparticles. Polyvinyl alcohol (PVA) is used as a surfactant in NP synthesis, which provides a negative charge and stabilizes the nanoparticles. We have prepared PLGA-Dtx nanoparticles with a mean diameter of 150 ± 10.2 nm and a polydispersity index close to 0.15. The polydispersity index was close to 0.1, indicating a limited size distribution and confirming the absence of agglomeration. The shape and surface morphology of PLGA-Dtx NPs were determined by using TEM and AFM. The surface morphology investigation employing TEM and AFM revealed spherical and homogeneous-sized nanoparticles. The AFM and TEM measurements revealed smaller particle diameters than the DLS measurements (90 ± 8.7 nm). This is due to the fact that the TEM and AFM observations of the nanoparticles were carried out while they were in their dry state form [35]. The serum proteins’ interaction with the PLGA-Dtx nanoparticles is the cause of the increase in particle size detected by DLS. In response to serum binding, the hydrophobic–hydrophobic interactions may lead the nanoparticles to aggregate. Size is a critical parameter while defining the delivery of nanoparticles in the test system. It has been demonstrated that nanoparticles with a size greater than 200 nm have the ability to become trapped in the reticuloendothelial system (RES), whereas particles with a size of 20 nm or less have the capacity to be eliminated from the body through the renal system [36]. The existence of a negative zeta potential makes it suitable for the delivery of NPs. The increased extent of hydrophobicity conferred by the phospholipid provides a solid core, which improves drug encapsulation efficiency. The in vitro release study of PLGA-Dtx indicated the sustained release till 72 h. This continuous release is attributable to the slow breakdown rate of polymers, and as a result, the release of docetaxel from the NPs relies primarily on drug diffusion and matrix erosion. This sustained release of docetaxel from PLGA nanoparticles may be useful for clinical use because it allows for drug delivery over a longer period of time while limiting drug-related systemic side effects.

Nonencapsulated chemotherapy medicines have previously been shown to selectively exert cytotoxic effects on tumor cells rather than normal cells [37]. Herein, we observed that PLGA-Dtx displays more powerful cytotoxic activity against A549 cells and PBMCs taken from lung cancer patients. On the other hand, they had lower cytotoxicity against normal human bronchial epithelial cells (BEAS-2B) and PBMCs derived from normal healthy donors. This is consistent with other studies, which have found anticancer activity of docetaxel nanoparticles against several cancer cell lines [38,39,40]. Additionally, our findings revealed that cancer cells were more prone to PLGA-Dtx uptake than normal cells. 

Further elaborating our findings, we explored the different endocytic pathways responsible for particle uptake by cancer cells using specific pathway inhibitors. Macropinocytosis, clathrin, and caveolae-mediated endocytosis may all exist in normal cells; however, macropinocytosis often acts synergistically with clathrin and caveolae-mediated endocytosis. Therefore, our results support prior studies indicating caveolae-mediated endocytosis is likely responsible for the internalization of PLGA-Dtx into cancer cells [40,41,42]. Caveolin-1, the key structural element of caveolae, is implicated in caveolae-mediated endocytosis. Despite the fact that the role of caveolin-1 in cancer is debatable, lung cancer has been shown to overexpress caveolin-1 [43]. Therefore, PLGA-Dtx may be internalized to greater extents in lung cancer cells than in healthy bronchial epithelial cells as a consequence of caveolae-mediated endocytosis. Altogether, these explicit features may facilitate cancer cells to improve nanoparticle uptake and elucidate the better cytotoxic results of PLGA-Dtx toward A549 cells. Though, advanced research is needed to better comprehend the mechanism related to PLGA-Dtx uptake in cancer cells.

Prior work using docetaxel suggested that mitotic arrest and necroptosis were induced in breast cancer cells when sensitizes to BAD. Docetaxel instigated synchronized plasma membrane damage and phosphatidylserine acquaintance on the surface and caused necroptotic cell death [14]. Apoptosis and necroptosis both were involved in the improved therapeutic potential of PLGA-Dtx in our study. The necroptosis inhibitor Nec-1 abolished PLGA-Dtx-induced cell death, whereas the apoptosis inhibitor Z-VAD-fmk partly reversed it. These data are in line with the study showing that necroptosis triggered cell death in L929 cells when cells were subjected to an apoptosis inhibitor [44]. This indicates the involvement of both cell pathways in the toxicity of PLGA-Dtx. Our findings might be useful in illustrating that when one mechanism of cell death is hindered, the other is triggered. The well-known apoptotic process is triggered by the activation of caspase-9 in reaction to the release of cytochrome c, which then leads to the activation of effector caspases including caspase-3, caspase-6, and caspase-7 [45]. In our study, we noticed an augmented expression of caspase-3, caspase-9, and proapoptotic protein Bax, as well as a decline in the expression level of the anti-apoptotic protein Bcl-2 and caspase-8. Moreover, molecules involved in necroptosis, such as RIP-1, RIP-3, and MLKL were upregulated by PLGA-Dtx treatment. Emerging findings suggest that reactive oxygen species (ROS) have a strong correlation with necroptosis; in addition, the manipulation of ROS is thought to be an effective treatment for cancer [46]. ROS and calcium signaling interact in a bidirectional manner; ROS may control cellular calcium signaling, while calcium signaling is necessary for the synthesis of ROS [47]. In current study, we also found that cytoplasmic calcium overload is caused by ROS. The major emphasis of earlier work on ROS was the cellular death that ROS produced. Albeit, fewer studies were focused on the relationship between ROS and necroptosis [48,49,50]. 

However, the direct effects of docetaxel on cancer cells alone cannot fully account for the potent cytotoxic activity. In fact, our studies with PBMCs derived from healthy donors showed that PLGA-Dtx treatment induces a robust cytokine response. The study demonstrated that PLGA-Dtx altered the relative percentage of CD3+, CD4+, and CD8+ T cells. Both docetaxel and paclitaxel seem to have immune stimulatory properties, according to various investigations [17,51,52]. Malignant cells and invading cells in many human malignancies produce and emit a variety of Th1, Th2, and Th17 cytokines (IL-1, IL-2, IL-4, IL-6, IL-10, IL-17, and IFN-γ) as well as TGF-β. IL-2, IL-4, and IFN-γ cytokines are primarily involved in the activation of cell and humoral immune responses, hence their increased levels in PLGA-Dtx-treated cells might suggest immune stimulation. The results are consistent with prior work that found that docetaxel and paclitaxel therapy resulted in a considerable rise in IFN- γ and IL-2 blood levels when compared with pretreatment baseline values [53]. The immunomodulatory effects seen might be attributed to the participation of necroptotic cell death, which is regarded as immunologically active. However, an in-depth study of necroptosis and confirmation of necroptotic-mediated immune responses was not possible in the current study. Further investigations are warranted with in vivo studies for confirmation.

## 5. Conclusions

Here, we have successfully synthesized and characterized PEGylated PLGA NPs encapsulating docetaxel for the treatment of lung cancers. The PLGA-Dtx NPs were uniformly spherical in shape and demonstrated sustained drug release for up to 72 h, enhancing the efficacy of tumor-targeted drug delivery while limiting side effects. The PLGA-Dtx NPs demonstrated improved selective cellular uptake in cancer cells and dramatically increased cytotoxicity against A549 cancer cells and PBMCs from cancer patients. Herein, we established that PLGA-Dtx may selectively persuade cell death in lung cancer cells compared with the free drug docetaxel alone. We also found that cell death persuaded by PLGA-Dtx treatment is not solely governed through apoptosis. We have shown that necroptosis, an alternative form of cell death, was also involved in cell death induced by PLGA-Dtx NPs. Consistently, PLGA-Dtx induced activation of the caspase pathway along with necroptosis (RIP1, RIP3, MLKL) more efficiently than the free drug. The findings revealed an increase in pro-inflammatory cytokines, which may be related to necroptosis. However, its correlation with necroptosis needs to be further proved and confirmed by a larger number of future studies. ROS generation, impaired mitochondrial membrane potential, and calcium influx were also implemented in PLGA-Dtx-mediated cell death. Our data suggest that PLGA-Dtx NPs may be a useful strategy for eliminating lung cancer cells. However, further in vivo studies are required to demonstrate the therapeutic efficacy and mechanistic aspects, particularly with regard to immune cells.

## Data Availability

The data presented in this study are available in this article.

## Supplementary Materials

The following supporting information can be downloaded at: https://www.mdpi.com/article/10.3390/vaccines10111801/s1, Figure S1: title; In vitro cytotoxicity of free Dtx and PLGA-Dtx NPs against A549 cancer cells at 48 h.
