# Supplementary material for: The Anti-Tumor and Immunomodulatory Effects of PLGA-Based Docetaxel Nanoparticles in Lung Cancer: The Potential Involvement of Necroptotic Cell Death through Reactive Oxygen Species and Calcium Build-Up
Source: Vaccines (Basel). 2022 Oct 26;10(11):1801. doi: 10.3390/vaccines10111801 (PMC9694303; doi:10.3390/vaccines10111801)
Supplement: Supplementary file 1 [file vaccines-10-01801-s001.zip › vaccines-1917154-supplementary.pdf]

Supplementary figure S1

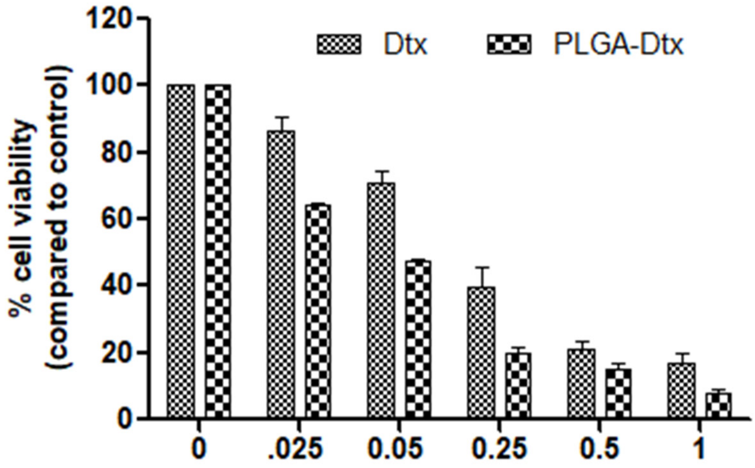

Figure S1. *In vitro* cytotoxicity of free Dtx and PLGA-Dtx NPs against A549 cancer cells at 48 h.
